# Case-finding for depression in primary care (CAIRO): a multicentre, cross-sectional study in England

**DOI:** 10.1136/bmjopen-2024-095040

**Published:** 2025-06-19

**Authors:** Sarah A Lawton, Christian Mallen, Carolyn Chew-Graham, Tom Kingstone, Sara Muller, Sarah Lewis, Ram Bajpai, Toby Helliwell

**Affiliations:** 1 Keele CTU, Keele University, Keele, UK; 2 School of Medicine, Keele University, Keele, UK; 3 Midlands Partnership University NHS Foundation Trust, Stafford, UK

**Keywords:** Primary Care, Depression & mood disorders, PUBLIC HEALTH, Patient Participation

## Abstract

**ABSTRACT:**

**Objectives:**

To examine the number of patients screening positive for depression, while self-completing an automated check-in screen prior to a general practice consultation.

**Design:**

A descriptive cross-sectional study.

**Setting:**

10 general practices in the West Midlands, England. Recruitment commenced in March 2023 and concluded in June 2023.

**Participants:**

All patients aged 18 years and over, self-completing an automated check-in screen for any general practice prebooked appointment, were invited to participate during a 3-week recruitment period.

**Primary and secondary outcome measures:**

The number of patients screening positive for depression using the Whooley case finding research questions was the primary outcome measure. Secondary outcome measures included: demographic and (general practice level) deprivation differences in completion responses.

**Results:**

73.5% (n=3666) of patients self-completing an automated check-in screen participated in the CAse-fInding foR depressiOn in primary care (CAIRO) study, (61.1% (n=2239) female, mean age 55.0 years (18–96 years, SD=18.5)).

28.3% (n=1039) of participants provided a positive response to at least one of the two Whooley research questions (31.2% female and 23.8% male). Significantly more positive responses were obtained from females, those aged between 35 years and 49 years and those from more deprived practices.

**Conclusions:**

Over a quarter of CAIRO participants provided a positive response to at least one of the two Whooley questions, suggesting possible unmet need in the population studied. A follow-up study could investigate whether responses provided at the point of check-in are raised and addressed in the subsequent consultation.

**STRENGTHS AND LIMITATIONS OF THIS STUDY**

- A rapid recruitment and participation method to offer case-finding for depression questions within general practice.
- Low-cost method of data collection for use by general practices to answer questions of relevance to them.
- Difficulty recruiting general practices to host the study.
- Not knowing if/how responses were considered as part of the pending consultation.

## Introduction

 Depression is a significant determinant of quality of life, a leading cause of disability and a major contributor to the disease burden worldwide.^1 2^ Depressive symptoms exist along a continuum from everyday sadness or distress^3^ to depression with suicidal ideation. General practitioners (GPs) have been found to be good at recognising moderate to severe depression,^4^ but they may be more likely to do so when patients present with psychosocial as opposed to somatic symptoms.^5 6^ A large WHO naturalistic study in 15 cities around the world (and in 11 languages) found that patients whose depression went unrecognised had milder symptoms at baseline and were not found to be at a disadvantage in terms of outcome.^7^ Studies since have suggested that a significant minority of people who might benefit from treatment remain undetected.^8 9^

The UK National Institute of Health and Care Excellence (NICE) recommends a matched-care model for the detection and management of depression.^10^ NICE guidance recommends that practitioners remain alert to the possibility of depression, especially in those with a previous history of depression and/or with a chronic physical health problem that affects their functioning. Population screening programmes have not been found to be cost-effective or to affect long-term health outcomes. One of the reasons that population screening is not recommended is that the number of false-positive responses is larger than the number of true-positive responses, due to the relatively low prevalence (<10%) of major depression among the primary care population.^11^ However, there has been no study to investigate the prevalence of depressive symptoms in a consulting primary care adult population: such adults are likely to have a higher likelihood of depression than the general population, particularly those patients consulting with long-term physical health conditions or those who are vulnerable.^12^ Depression is a significant cause of global morbidity, and while screening and case-finding are promoted in many high-income countries, this is not standard practice. The evidence base for the detection of depression in primary care settings in low and middle-income countries is limited.^13^

The barriers to people with long-term health conditions discussing mood problems in consultations are well-described.^14^ There is increasing evidence to show that if depression is detected and managed in people with long-term conditions and in older adults, then a range of patient outcomes can be improved^15 16^ and the requirement for medication to treat depressive symptoms will be decreased. Depression and multimorbidity (two or more long-term conditions) are more common in areas of socioeconomic deprivation, which may be inner city or rural.^17^

The validated Whooley research questions concerning mood and interest can be used as case-finding questions,^18^ specifically: ‘*During the past month, have you often been bothered by feeling down, depressed or hopeless?*’ and ‘*During the past month, have you often been bothered by having little interest or pleasure in doing things?*’. These two ‘Whooley’ questions are highly sensitive for identifying depression,^19^ and thus responding in the negative to these items can be taken to indicate that the patient does not have depression.^20^ A positive response to one or both questions does not indicate a diagnosis of depression but indicates that further exploration is needed.

This study examined the number of patients screening positive for depression, while self-completing an automated check-in screen prior to any general practice consultation. The association of demographic factors and (general practice level) deprivation was also considered in relation to positive screening for depression.

## Methods

### Design

The use of automated check-in screens to collect brief research data has been previously investigated (the ‘automated check-in data collection (AC DC) methodology’).^21^ On entering general practice premises, patients independently approach a check-in screen, touch the screen to select successively their sex and their day and month of birth. This then confirms the patient’s arrival for their consultation. Use of the screen to check-in is encouraged in all general practices but is optional, and there is a reception desk available in all practices as an alternative. Where available, some check-in screens have the facility to collect additional brief data from the patient, adding responses automatically to the patient’s medical record.

16 general practices within the National Institute for Health and Care Research (NIHR) Clinical Research Network (CRN): West Midlands (an organisation with responsibility for facilitating research in the English National Health Service), whose General Practice Information Technology systems and services (GPIT Futures) were from EMIS Health (formerly known as Egton Medical Information Systems), was invited to host the ‘CAse-fInding foR depressiOn in primary care’ (‘CAIRO’) study. Participating general practices had access to Egton Automated Arrival facilities, to include an automated arrivals check-in screen and a Questionnaire Module.

### Participants

During the 3-week recruitment period in each practice, all patients 18 years of age and older, who were attending a booked appointment and completed an automated check-in screen to confirm their attendance for their appointment, were eligible to participate. Patient-facing documentation, which included an invitation poster and a participant information leaflet, was available next to the automated check-in screen. Once a patient had confirmed their attendance for a booked appointment, the research questions appeared on the screen for completion. The research questions did not appear on any subsequent appointment check-in screen during the recruitment period for those who had already responded to the research questions.

### Consent

Regulatory approvals were obtained based on implied consent for anyone of age 18 years and older, due to the rapid way in which this study was conducted and in line with the definition outlined in Article 4 (11) of the General Data Protection Regulation guidance.^22^ Participants were provided with up to 4 weeks, a ‘cooling off’ period (before pseudonymised data were downloaded), should they wish to retract or amend their participation in the study.

### Data collection

This study was granted regulatory approvals on 18 November 2019; however, before the study could start recruitment, the emergence of the COVID-19 pandemic halted study commencement. The number of patients physically attending general practice declined, and all high-intensity physical touchpoints were removed from public areas, including check-in screens. Furthermore, COVID-19 related research was prioritised by the NIHR CRN. The research team monitored this situation and restarted the study when face-to-face appointments as standard practice and the use of automated check-in screens were reintroduced.

Participating practices collected data during a 3-week period, between March and June 2023. The validated Whooley^18^ questions used were: ‘*During the past month, have you often been bothered by feeling down, depressed or hopeless?’*, with responses: *‘Yes’, ‘No’* or *‘Skip’*, and then, ‘*During the past month, have you often been bothered by having little interest or pleasure in doing things?’*, with responses: *‘Yes’, ‘No’* or ‘*Skip’*. Responses were automatically filed back to the patients’ electronic medical record, and if a ‘*Yes’* response had been provided to at least one of the two questions, the following message was displayed: *‘As you have answered ‘Yes’ to at least one of these questions, please consider talking to your GP about how you feel’.*

A series of pseudonymised data extractions following recruitment were conducted by participating practices, and data were securely transferred to the research team for analysis.

### Patient and public involvement and engagement

A patient and public involvement and engagement (PPIE) group of 8 people from Keele University’s research user group (RUG) was convened to assist in designing and developing the study. The patient-facing documentation was codeveloped with the PPIE group. They were asked to consider the patient-facing documents in terms of content, layout, wording, style and length. Recommendations for dissemination of findings were discussed by the group.

### Data analysis

Descriptive statistics (mean and SD or median with IQR for continuous variables and frequency with percentage (%) for categorical variables) were used to characterise the study sample and to compare potential demographic differences between responders and non-responders. A χ^2^ test was used to make statistical comparisons between groups as appropriate. Two-sided p value < 0.05 was considered for statistical significance. IBM SPSS Statistics V.29.0 (Armonk, New York, USA) statistical software was used for data analysis. In the production of and reporting on subgroups (practice, age group, sex), cell counts <5 were suppressed to ensure that the confidentiality of individual persons was protected.^23^

## Results

10 general practices, out of the 16 approached, with a total population of 81 285 people, aged 18 years of age or over, agreed to host the study. Over the 3-week recruitment window, practices were live to recruitment for an average (median) of 13.5 days (IQR 13–14 days, range 4–15 days). Variations were due to bank holidays causing general practice closures and the one practice ceasing recruitment after just 4 days.

**Figure 1:**
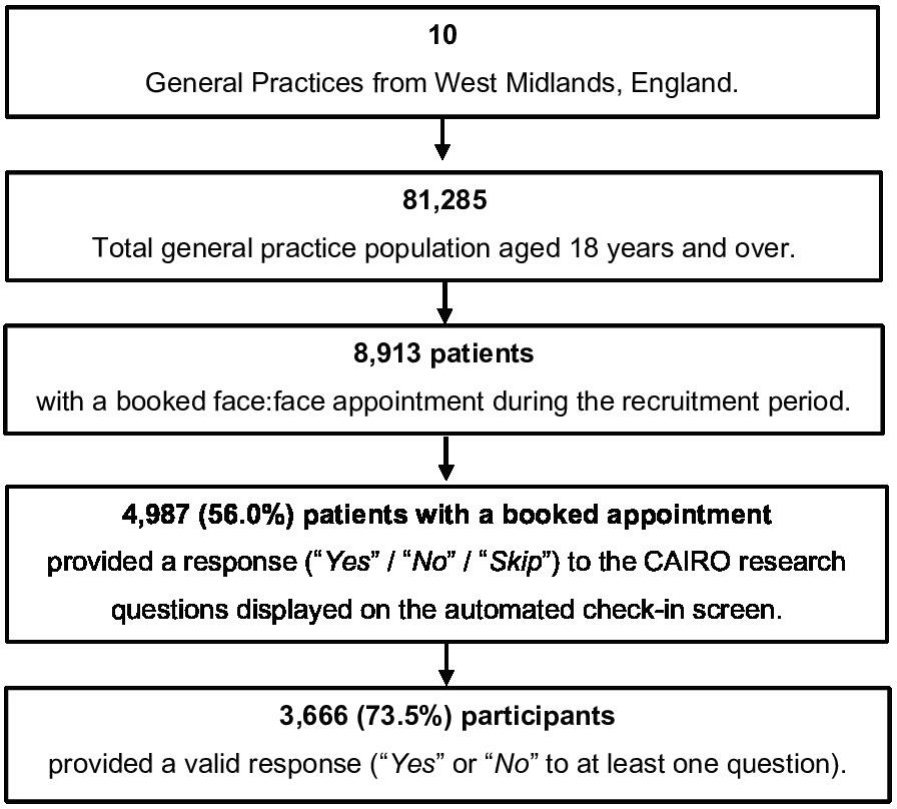
Summary of CAIRO study participants.

Participating practice deprivation, based on the 2019 English Index of Multiple Deprivation deciles,^24^ ranged from 2 to 10. Deprivation decile 1 is most deprived and 10 is least deprived. The last practice completed data collection on 30 June 2023. There were 8913 eligible patients with a booked face-to-face appointment during the study recruitment period. 4987 (56.0%) patients with a booked appointment provided a response to the research questions displayed on the automated check-in screen. Of these, 3666 (73.5%) provided a valid response to the research questions (figure 1). The remainder chose the ‘*Skip*’ option for both questions.

CAIRO study population demographics are summarised in table 1.

**Table 1:** CAIRO study population demographics

	Age (years)	Sex (female)
	Mean (SD)	N (%)
Demographics of those with a booked appointment n=8913	55.8 (19.2)	5462 (61.3)
Demographics of those responding to the research questions n=4987	53.6 (18.6)	3150 (63.2)
Demographics of those providing a valid response to the research questions (participants) n=3666	55.0 (18.5)	2239 (61.1)

CAIRO, CAse-fInding foR depressiOn in primary care.

The mean age of those with a booked appointment was slightly higher than the mean age of those responding to the research questions. The mean age of those with a booked appointment and participants, though, was similar (55.8 years vs 55.0 years). The percentage of females with a booked appointment and the percentage of female participants were both lower than the percentage of females responding to the research question.

### CAIRO research question valid responses

Of the 3666 participants providing at least one valid response to the Whooley questions, 3500 (95.5%) participants responded *‘Yes’* or *‘No’* to the first question, *‘During the past month, have you often been bothered by feeling down, depressed or hopeless?’*. 3106 (84.7%) participants responded ‘*Yes’* or *‘No’* to the second question, ‘*During the past month, have you often been bothered by having little interest or pleasure in doing things?’*. The combination of valid answers provided is displayed in table 2.

**Table 2:** CAIRO responses to the Whooley questions

		Q1 “During the past month, have you often been bothered by feeling down, depressed or hopeless?					
		‘Yes’		‘No”’		‘Skip’	
		n	%	n	%	n	%
**Q2 ‘During the past month, have you often been bothered by having little interest or pleasure in doing things?‘**	**‘Yes’**	700	19.1%	81	2.2%	64	1.7%
	**‘No’**	100	2.7%	2059	56.2%	102	2.8%
	**‘Skip’**	94	2.6%	466	12.7%		

CAIRO, CAse-fInding foR depressiOn in primary care.

1039 (28.3%) participants responded *‘Yes*’ to at least one of the two Whooley questions potentially indicating depression. These participants are compared with those participants that provided other combinations of responses (n=2627) by practice, practice deprivation, participant age and gender, in table 3.

**Table 3:** Characteristics of participants who did and did not screen positive for potential depression

	CAIRO research question response:	
	Included a ‘yes’ response	Did not include a ‘yes’ response
	% (n)	% (n)
**Practice** (p value ≤0.001)		
1	30.0% (165)	70.0% (385)
2	35.8% (53)	64.2% (95)
3	19.8% (51)	80.2% (207)
4	30.8% (84)	69.2% (189)
5	21.1% (139)	78.9% (521)
6	36.1% (123)	63.9% (218)
7	35.0% (70)	65.0% (130)
8	35.0% (117)	65.0% (217)
9	32.6% (94)	67.4% (194)
10	23.3% (143)	76.7% (471)
** Totals**	**28.3% (1039)**	**71.7% (2627)**
Practice IMD decile* (p value ≤0.001)		
≤5	32.9% (653)	67.1% (1333)
>5	23.0% (386)	77.0% (1294)
** Total**	**28.3% (1039)**	**71.7% (2627)**
**Age group** (years) (p value ≤0.001)		
18–34	32.0% (216)	68.0% (459)
35–49	35.3% (246)	64.7% (451)
50–64	31.8% (308)	68.2% (661)
65–79	21.5% (222)	78.5% (809)
80+	16.0% (47)	84.0% (247)
** Totals**	**28.3% (1039)**	**71.7% (2627)**
**Sex** (p value ≤0.001)		
Female	31.2% (699)	68.8% (1540)
Male	23.8% (340)	76.2% (1087)
** Total**	**28.3% (1039)**	**71.7% (2627)**

* Deprivation was based on the 2019 English IMD,^24^ using population weighted GP deprivation scores, where a practice deprivation score of 1 means most deprived and a deprivation score of 10 means least deprived.

GP, general practitioner; IMD, Index of Multiple Deprivation.

Those participants that responded *‘Yes*’ to at least one of the research questions (n=1039) varied significantly by practice and practice-level deprivation score (depression was more likely from participants registered in more deprived practices). In addition, more females provided a *‘Yes*’ response than males (31.2% vs 23.8%) and those aged 35–49 years were more likely to provide a *‘Yes*’ response, with the proportion of participants indicating symptoms of depression reducing as age increased.

## Discussion

This study has examined the proportion of consulting primary care patients screening positive for depression, while self-completing an automated appointment check-in screen. 56.0% of patients with a booked appointment during the recruitment period provided a response to the CAIRO research questions. 73.5% (3666) of these responses provided valid participation in the study (discounting *‘Skip’, ‘Skip’* responses). This is lower than the response rates observed in previous studies conducted by the research team using automated check-in screens or the AC DC methodology, 85.1%^21^ and 80.3%.^25^ Reasons for this could include a reluctance to use the automated check-in screens as a touch point following the COVID-19 pandemic; the topic of the research question; perspectives on research; or not having the time to complete. 26.5% of those patients responding to the research questions selected the *‘Skip’, ‘Skip*’. This was not a valid response, making them ineligible for participation. It also highlights a limitation of this method, in that it is not possible to capture reasons for non-completion.

28.3% of participants in the CAIRO study provided a positive response to at least one of the two Whooley questions. The prevalence of depression increased and did so particularly among vulnerable adults during the COVID-19 pandemic.^12^ Females and those participants in the 35–49-year age range were most likely to provide a positive response to the Whooley questions in the CAIRO study. These findings correlate with the WHO data 2023.^26^ The variation by practice in the percentage of participants providing a positive response to at least one of the two Whooley questions also suggests that deprivation is a contributory factor,^27^ with more deprived practice populations providing a significantly higher percentage of positive responses. Variations may also be due to practice location (rural vs urban) or the nature of the support services provided by the general practice already. The significant differences identified in different demographic groups provide opportunities for further research and identify areas of potential unmet need.

Use of the AC DC methodology has provided an opportunity to collect data rapidly, while also updating patients’ medical records with answers to the two validated Whooley questions. There was a prompt for patients responding positively to the research question(s), asking them to consider discussing how they are feeling with the GP. A limitation of this study was that we could not determine the proportion of people who then did so. The recording of the responses to the research questions does, though, provide information to aid future primary care contacts.

16 general practices were invited to host the research study. Six practices declined to participate due to a concern about the number of patients potentially providing a positive indication of depression. While this study was eligible for proportionate ethical review, the study team was invited to discuss the application with the research ethics committee (REC). They too were concerned that positive responses to the research questions could increase practice workload. The concerns highlighted by the REC, the reluctance of the practices to host the research study, to include one practice withdrawing soon after starting, together with a drop in the number of patients providing a valid response to this type of study, suggests that the AC DC methodology may be better at collecting data on some topics rather than others.

### Strengths of the CAIRO study

Using the AC DC methodology has again provided a cost-effective and convenient way (where the required digital tools exist) to collect brief research data. In this study, data have been collected on patients’ mood, updating the medical record with responses to the two Whooley questions, which can then be used to initiate further exploration of depressive symptoms.^10^ The method does not require any researcher-participant interaction. The automated delivery of the study allows remote set-up, little disruption to the general practice and ensures that sampling bias is minimised, with delivery of the study remaining consistent. The technology used also enables the study team to remotely conduct real-time monitoring of the data being collected. This continues to provide an efficient model for embedding research into the general practice setting. The method enables population-specific sampling and associated clinical coding into the electronic medical record.

Codeveloping the patient-facing documentation with the Keele University RUG enhanced the clarity of information and supported acceptability of the data being collected. Future studies using the AC DC methodology should work with relevant patient groups and RECs to ensure and validate appropriate presentation of the research questions, functioning logic and response options.

### Limitations of the CAIRO study

44.0% of those with a booked appointment did not provide a response to the research questions. Collecting data using the AC DC methodology^21^ does not provide any context, with only a limited amount of data being collected. While 26.5% of those responding to the research questions answered *‘Skip’*, others may have simply waited until the screen cleared and returned to the home menu. It was not possible to obtain any information on why these patients did not wish to participate in the study. Response rates to this method of invitation to participate in research, however, are higher than other methods observed from alternative more traditional methods of invitation.^28^ A proportion of patients will have also checked in for their appointment with the receptionist, due to needing to discuss other matters, having visual impairments, language difficulties or feeling too unwell to use the screen. Higher participation in the research may have been achieved if patients were facilitated or encouraged to use the check-in screen to confirm their attendance for their booked appointment.

For those patients responding *‘Yes’* to either of the research questions, a prompt appeared, asking them to consider discussing how they were feeling with the GP. In what proportion of cases this was actioned, though, was not investigated in this study. Similarly, whether the consulting practitioner discussed the patient’s responses to the research questions with the patient, whether the responses have aided future consultations or whether the patient was already known to experience depressive symptoms is also unknown.

While some sampling biases are minimised by use of the automated check-in technology, other sampling biases are introduced. Only those general practices whose GPIT Futures was EMIS Health and only those patients with a booked face-to-face appointment in the recruitment period and choosing to confirm their attendance for their booked appointment by using the check-in screen were invited to participate. While only a minority of patients registered with each practice were eligible to participate in the study, as the recruitment window was only 3 weeks, further selection of the participating group may have occurred by their ability to use the screen technology. Similar to previous studies using this methodology,^21 25^ those taking part in the CAIRO study had a similar age and gender distribution to all those with booked appointments during the recruitment period.

A positive response to at least one of the two Whooley questions does not indicate a diagnosis of depression but indicates that further exploration is needed. Those patients identified in this study as needing further exploration may already have symptoms known to the practice. In some cases, however, this approach to case finding may be useful when coupled with an additional follow-up measure to ensure depression does not go undetected.

## Conclusion

Over a quarter of CAIRO participants provided a positive response to at least one of the two Whooley questions. This suggests a possible unmet need in the population studied. It is important to distinguish distress from depression,^29^ so that appropriate management options are offered to people with the range of depressive symptoms reported.^10^ Increasing evidence shows that if depression is detected and appropriate management offered, a range of patient outcomes can be improved.^30^ A follow-up study could investigate whether responses provided at the point of check-in are then addressed in the subsequent consultation or whether information about mental health problems and sign-posting can be included in the check-in process for people who respond positively to the case-finding questions. In addition, recent findings have demonstrated that the use of digital health tools can also be effective in reducing the symptoms of depression.^31^

While more case-finding activities for depression should be supported within primary care, doing this at the check-in screen cannot be yet recommended. There is a need to first explore reasons around reluctance by practices to investigate this area. It may be true that there was reluctance by patients to respond to such case-finding questions; however, the reluctance could also have been due to a range of other more practical factors. With further acceptability testing, the AC DC methodology still offers great potential to capture time-sensitive health data at large scale. Potential use of the check-in screens for health promotion activity, vaccination invitations and alerting to services is also another area of investigation for future research.

## Data Availability

Data are available upon reasonable request.
