# Supplementary material for: Case-finding for depression in primary care (CAIRO): a multicentre, cross-sectional study in England
Source: BMJ Open. 2025 Jun 19;15(6):e095040. doi: 10.1136/bmjopen-2024-095040 (PMC12182143; doi:10.1136/bmjopen-2024-095040)
Supplement: Supplementary file 1 [file bmjopen-2024-095040.reviewer_comments.pdf]

# PEER REVIEW HISTORY

BMJ Open publishes all reviews undertaken for accepted manuscripts. Reviewers are asked to complete a checklist review form and are provided with free text boxes to elaborate on their assessment. These free text comments are reproduced below.

## ARTICLE DETAILS

**Title (Provisional)**

Case-finding for depression in primary care (CAIRO): a multicentre, cross-sectional study in England

**Authors**

Lawton, Sarah Ann; Mallen, Christian; Chew-Graham, Carolyn; Kingstone, Tom; Muller, Sara; Lewis, Sarah; Bajpai, Ram; helliwell, toby

---

## VERSION 1 - REVIEW

---

| | |
|---|---|
| **Reviewer** | **1** |
| **Name** | **Holst, Anna** |
| **Affiliation** | **Primary Health Care, School of Public Health and Community Medicine, Institute of Medicine, Sahlgrenska Academy, University of Gothenburg** |
| **Date** | **11-Dec-2024** |
| **COI** | **None** |

---

Thank you for the opportunity to review your manuscript. It is well-written and easy to understand. However, y most important input, I noticed a lack of information regarding the overall check-in process at the practices. Is self-check-in optional, with alternative options available, such as assistance from a receptionist at a desk? This context is important, and it would be helpful if you could include the proportion of patients who opted for self-check-in when other options were available.

A few other thoughts:

The results section:

It is well-organized but would benefit from additional narrative explanations of the findings presented in the tables. For example, the authors could emphasize the most significant differences between demographic groups and explain their practical relevance.

Exploration of reasons for non-participation:

The manuscript notes that 26.5% of patients chose "Skip," and 44% did not participate. Expanding on potential reasons for this non-participation (e.g., technical difficulties,

sensitivity surrounding the topic) would be helpful. Additionally, suggestions for overcoming these challenges in future studies could strengthen the discussion.

Clarification of practical implications:

While the study identifies a potentially high prevalence of depressive symptoms, it remains unclear how these findings could be integrated into clinical practice. The authors might consider elaborating on how healthcare providers could utilize this data, particularly given the lack of follow-up on whether patients discussed their responses with their doctors.

Methodological clarifications:

The authors acknowledge that selection bias may have occurred due to limited access to check-in screens and varying technological skills among participants. A more detailed analysis of how this might affect the study's generalizability would provide added depth to the discussion.

Consider a more explicit conclusion:

The conclusion is appropriately cautious but could benefit from a more definitive summary of the study's significance for both future research and clinical practice. A stronger emphasis on the implications of the findings would enhance the impact of the manuscript.

Minor language adjustments:

The manuscript is generally well-written, but a few sections could be revised for greater clarity and conciseness. For instance, breaking up longer sentences in the discussion section would improve readability and comprehension.

Statistical analysis clarifications:

Although the statistical methods are adequately described, providing further interpretation of why certain age and gender groups exhibited higher rates of positive responses would help contextualize the findings.

Figures and tables:

The tables are effective, but adding a visual element, such as a chart or graph illustrating differences in positive responses across demographic groups, could enhance accessibility and engagement for readers.

---

| | |
|---|---|
| **Reviewer** | **2** |
| **Name** | **Zoromba, Mohamed** |
| **Affiliation** | **Mansoura University Faculty of Nursing** |
| **Date** | **17-Jan-2025** |
| **COI** | **None** |

---

This manuscript presents an important study on case-finding for depression in primary care using automated check-in screens. The study is well-structured, but there are areas where clarity, depth, and alignment with existing literature can be improved. Minor revisions are recommended to address these issues and enhance the manuscript's overall impact.

Abstract

Results Presentation: Include numerical results, such as the proportion of participants screening positive for depression by demographics. (Page 2, Lines 8–15)

Introduction

Contextualization: Broaden the discussion to include global perspectives on depression detection in primary care, particularly in similar healthcare systems. (Page 4, Lines 20–35)

Methods

Bias and Limitations: Expand on how biases related to self-selection and use of automated screens were mitigated. (Page 7, Lines 15–30)

Deprivation Index: Clarify how the deprivation index was operationalized in practice-level analyses. (Page 8, Lines 5–15)

Results

Clarity in Data Presentation: Reorganize Table 3 to emphasize significant findings, such as the relationship between deprivation and positive responses. (Page 11, Lines 10–25)

Discussion

Integration with Literature: Strengthen the discussion by comparing findings to recent studies on digital tools in primary care and depression case-finding. (Page 14, Lines 20–35)

Practical Implications: Offer more detailed recommendations for integrating automated check-in tools into clinical workflows. (Page 15, Lines 10–20)

Limitations

Non-responders: Discuss potential reasons for non-response and their impact on generalizability. (Page 16, Lines 15–30)

To contextualize the study further, consider citing the following research:

Effects of Emotional Intelligence Training on Symptom Severity in Patients With Depressive Disorders, Advancing trauma studies: A narrative literature review embracing a holistic perspective and critiquing traditional models, The Mediating Role of Psychological Capital in the Relationship Between Family Sense of Coherence and Caregiver Stress Among Parents of Children With Autism Spectrum Disorder, The Role of Psychological Ownership in Linking Decent Work to Nurses' Vigor at Work.

## VERSION 1 - AUTHOR RESPONSE

Reviewer: 1
**Dr. Anna Holst, Primary Health Care, School of Public Health and Community Medicine, Institute of Medicine, Sahlgrenska Academy, University of Gothenburg**

**Comments to the Author:**

Thank you for the opportunity to review your manuscript. It is well-written and easy to understand. However, y most important input, I noticed a lack of information regarding the overall check-in process at the practices. Is self-check-in optional, with alternative options available, such as assistance from a receptionist at a desk? This context is important, and it would be helpful if you could include the proportion of patients who opted for self-check-in when other options were available.

**Thank you for this comment and we agree context of the check-in process at practices is important. In the 'Methods' section and sub-section 'Design', we do refer readers to a paper (https://pubmed.ncbi.nlm.nih.gov/36604124/) which outlines detail on the use of automated check-in screens to collect brief research data. Without wanting to repeat the detail described in this referenced paper, within this section, we have now also included the statement, "Use of the screen to check-in is encouraged in all general practices but optional, and there is a reception desk available in all practices as an alternative*". The 'Discussion' section, in sub-section 'Limitations of the CAIRO study', also explains that some patients *"will have also checked in for their appointment with the receptionist, due to needing to discuss other matters, having visual impairments, language difficulties or were feeling too unwell to use the screen".***

**Figure 1 highlights that 4,987/8913 (56.0%) of those with a booked face-to-face appointment provided a response ("*Yes*" / "*No*" / "*Skip*") to at least one of the two research questions displayed. Unfortunately, we did not have a report to detail the number using the screen to check-in and not responding to the research questions displayed**.

A few other thoughts:
The results section:
It is well-organized but would benefit from additional narrative explanations of the findings presented in the tables. For example, the authors could emphasize the most significant differences between demographic groups and explain their practical relevance.

**Thank you for this comment, we agree that highlighting the most significant differences between demographic groups and their relevance is important. Both the 'Results' and 'Discussion' sections have been updated to highlight that, *"more females provided a "Yes" response than males (31.2% versus 23.8%)"* and *"The significant differences identified in different demographic groups provide opportunities for further research and identify areas of potential unmet need."***

Exploration of reasons for non-participation:
The manuscript notes that 26.5% of patients chose "Skip," and 44% did not participate. Expanding on potential reasons for this non-participation (e.g., technical difficulties, sensitivity surrounding the topic) would be helpful. Additionally, suggestions for overcoming these challenges in future studies could strengthen the discussion.

**Whilst it was not possible to obtain any information on why these patients did not wish to participate in the study, further discussion has been included in the paper (page 14), with a suggestion on how to overcome the challenge of participation.**

Clarification of practical implications:
While the study identifies a potentially high prevalence of depressive symptoms, it remains unclear how these findings could be integrated into clinical practice. The authors might consider elaborating on how healthcare providers could utilize this data, particularly given the lack of follow-up on whether patients discussed their responses with their doctors.

**We agree that this would be interesting to explore further in future research to establish whether responses provided at the point of check-in, are then addressed in the subsequent consultation; how healthcare providers could best utilise the data provided at the point of check-in; whether a follow-up separate to their reason for consultation (if it was not to discuss their mental health) should be provided; whether further information and sign-posting to mental health resources could be provided to patients answering 'Yes' would be appropriate. Such suggestions are included in the 'Discussion' and 'Conclusion' sections of the manuscript.**

Methodological clarifications:
The authors acknowledge that selection bias may have occurred due to limited access to check-in screens and varying technological skills among participants. A more detailed analysis of how this might affect the study's generalizability would provide added depth to the discussion.

**We appreciate your concern about the generalisability of results. We have explored options for detailed analysis to provide generalisable results however, we're limited, with only the background variables as presented in the table 3 and therefore, we only attempted to conduct exploratory analysis. We had added and discussed this point in the limitations.**

**In considering this further, we think this point should be considered more by, implications of any selection bias. So for example, are people who don't complete the check-in screen due to their lack of technical skills, more likely to be depressed?, would depression encourage or discourage use of the screen in favour of the reception desk? And the answer to these questions we can't answer from this study alone.**

Consider a more explicit conclusion:
The conclusion is appropriately cautious but could benefit from a more definitive summary of the study's significance for both future research and clinical practice. A stronger emphasis on the implications of the findings would enhance the impact of the manuscript.

**Thank you for this comment. With the limited data available from this study, finding implications for depression are limited, however we have added further research ideas to the 'Conclusion' section. We have also added additional ideas for the use of the check-in screens, for future research. Implications of which, could be much wider than this investigation into case-finding for depression.**

Minor language adjustments:
The manuscript is generally well-written, but a few sections could be revised for greater clarity and conciseness. For instance, breaking up longer sentences in the discussion section would improve readability and comprehension.

**Thank you. This has been addressed.**

Statistical analysis clarifications:
Although the statistical methods are adequately described, providing further interpretation of why certain age and gender groups exhibited higher rates of positive responses would help contextualize the findings.

**Evidence is that females more likely to be diagnosed with depression than males and older adults less likely to admit to low mood due to stigma. We have signposted this correlation in findings to the World Health Organisation data, on page 12.**

Figures and tables:
The tables are effective, but adding a visual element, such as a chart or graph illustrating differences in positive responses across demographic groups, could enhance accessibility and engagement for readers.

**Thank you for your excellent suggestion and we explored options like two-way bar diagram. However, we find it easier with the table to show all results at one place. Therefore, we'd like stick with the current tables but do really appreciate your suggestion.**

**<u>Reviewer: 2</u>**
**Dr. Mohamed Zoromba, Mansoura University Faculty of Nursing**

**Comments to the Author:**
This manuscript presents an important study on case-finding for depression in primary care using automated check-in screens. The study is well-structured, but there are areas where clarity, depth, and alignment with existing literature can be improved. Minor revisions are recommended to address these issues and enhance the manuscript's overall impact.

Abstract
Results Presentation: Include numerical results, such as the proportion of participants screening positive for depression by demographics. (Page 2, Lines 8–15)

**Thank you for your positive comments. We have updated Results as requested.**

Introduction
Contextualization: Broaden the discussion to include global perspectives on depression detection in primary care, particularly in similar healthcare systems. (Page 4, Lines 20–35)

**Thank you for this helpful suggestion. The discussion has been broadened on page 4.**

Methods
Bias and Limitations: Expand on how biases related to self-selection and use of automated screens were mitigated. (Page 7, Lines 15–30)

**Biases introduced through, only those patients choosing to confirm their attendance for their booked appointment by using the check-in screen, being able to participate were not mitigated**

**against. It has been suggested that patients could have been facilitated to or encouraged to use the check-in screen to confirm their attendance for their booked appointment. Doing this, however, would not reflect a pragmatic approach to activity within a usual general practice setting. We did not want to interfere with normal practice, as we were interested in who would be picked up by the screening in this way.**

Deprivation Index: Clarify how the deprivation index was operationalized in practice-level analyses. (Page 8, Lines 5–15)

**Participant deprivation was not available. Practice deprivation was therefore taken as a crude estimate for its registered population. Further detail around this has been provided.**

Results
Clarity in Data Presentation: Reorganize Table 3 to emphasize significant findings, such as the relationship between deprivation and positive responses. (Page 11, Lines 10–25)

**We apologise for the confusion. Table 3 is showing an exploratory analysis of potential association between CAIRO research question response on screen positive for potential depression (yes or no) and background variables. We presented this data with a focus on who responded "Yes" in each category, and overall p-value indicating significant association between the background variable and CAIRO research question response. We hope this clarifies your comment.**

Practical Implications: Offer more detailed recommendations for integrating automated check-in tools into clinical workflows. (Page 15, Lines 10–20)

**Thank you for this comment. A recommendation to improve participation has been noted in the 'Discussion' section.**

Limitations
Non-responders: Discuss potential reasons for non-response and their impact on generalizability. (Page 16, Lines 15–30)

**Thank you for your comment. We have explored options for detailed analysis to provide generalisable results however, we're limited, with only the background variables as presented in the table 3 and therefore, we only attempted to conduct exploratory analysis. We had added and discussed this point in the limitations.**

To contextualize the study further, consider citing the following research:
Effects of Emotional Intelligence Training on Symptom Severity in Patients With Depressive Disorders, Advancing trauma studies: A narrative literature review embracing a holistic perspective and critiquing traditional models, The Mediating Role of Psychological Capital in the Relationship Between Family Sense of Coherence and Caregiver Stress Among Parents of Children With Autism Spectrum Disorder, The Role of Psychological Ownership in Linking Decent Work to Nurses' Vigor at Work.

**Thank you for your comment. We have looked at this paper and do not feel it is relevant to this manuscript.**
